# Temporal dynamics of urban gas pipeline risks

**DOI:** 10.1038/s41598-024-56136-9

**Published:** 2024-03-06

**Authors:** Fatema Rahimi, Abolghasem Sadeghi-Niaraki, Mostafa Ghodousi, Tamer Abuhmed, Soo-Mi Choi

**Affiliations:** 1 Department of Computer Science and Engineering and Convergence Engineering for Intelligent Drone, XR Research Center, Sejong University, Seoul, Republic of Korea; 2 Geoinformation Technology Center of Excellence, Faculty of Geodesy and Geomatics Engineering, K.N. Toosi University of Technology, Tehran, Iran; 3 College of Computing and Informatics, Sungkyunkwan University, Suwon, 16419 Republic of Korea

**Keywords:** Day–night population distribution, Vulnerability assessment, Hazard assessment, Urban gas pipeline risks, Environmental impact, Natural hazards

## Abstract

Urban gas pipelines pose significant risks to public safety and infrastructure integrity, necessitating thorough risk assessment methodologies to mitigate potential hazards. This study investigates the dynamics of population distribution, demographic characteristics, and building structures to assess the risk associated with gas pipelines. Using geospatial analysis techniques, we analyze population distribution patterns during both day and night periods. Additionally, we conduct an in-depth vulnerability assessment considering multiple criteria maps, highlighting areas of heightened vulnerability in proximity to gas pipelines and older buildings. This study incorporated the concept of individual risk and the intrinsic parameters of gas pipelines to develop a hazard map. Hazard analysis identifies areas with elevated risks, particularly around main pipeline intersections and high-pressure zones. Integrating hazard and vulnerability assessments, we generate risk maps for both day and night periods, providing valuable insights into spatial risk distribution dynamics. The findings underscore the importance of considering temporal variations in risk assessment and integrating demographic and structural factors into hazard analysis for informed decision-making in pipeline management and safety measures.

## Introduction

The demand for natural gas as an adaptable energy source for high-value products has grown significantly, especially in the industrial and petrochemical sectors. Although the expansion of gas transmission lines has brought economic benefits through energy supply and exports, it has also raised environmental and safety concerns^1–3^. Accidents involving natural gas pipelines, although infrequent, have the potential for extensive devastation that involves pollution, injuries, property damage, hospitalizations, fatalities, and large-scale evacuations^4–6^. Given the potential hazards associated with urban gas pipelines, it is imperative to implement effective risk assessment methodologies to identify, evaluate, and mitigate potential risks. Comprehensive risk assessments consider various factors, including population dynamics, demographic characteristics, building structures, and pipeline parameters, to provide insights into spatial risk distribution and inform decision-making in pipeline management and safety measures^7^.

To address these complex challenges, the multifaceted factors that contribute to these incidents must be understood. Previous research in this field has focused on three key areas, each of which provides valuable insights into urban gas pipeline risks. The first area focuses on spatiotemporal population modeling, with studies aimed at predicting population distribution changes and dynamic estimates of population density. For instance, Freire and Aubrecht^8^ predicted the population distribution in Lisbon using land use data to account for the differences between daytime and nighttime populations. Aubrecht et al.^9^ investigated the impact of population distribution dynamics on crisis management by utilizing land use data. Renner et al.^10^ enhanced risk assessment in tourist areas by modeling population changes in Bolzano at different times. Ma et al.^11^ modeled the hourly population distribution in Beijing by employing a combination of census data, subway passenger numbers, and geospatial data. Li et al.^12^ investigated spatiotemporal population distribution by utilizing heat maps and points of interest data. Xia et al.^13^ used call record data to design a population density model for real-time seismic zone population estimates. Most of these studies utilized land use data, which have been used to improve the accuracy of population distribution models, particularly in countries where only census data are available.

The second area covers gas pipeline risk assessment, which involves the assessment of the probability of gas pipeline failures, their consequences, and the associated individual and social risks, as done by Ma et al.^14^ using quantitative methods. Amir-Heidari et al.^15^ assessed the individual and social risks associated with Iran’s natural gas distribution network, considering fire and explosion scenarios. Bariha et al.^16^ analyzed the hazards of natural gas and oil pipelines, aiming to provide a mathematical model for evaluating gas release and hazard distances, particularly in the event of high-pressure gas release. Azari and Karimi^17^ investigated urban gas pipeline risk by combining a hazard map with a vulnerability map to evaluate gas pipeline hazards, emphasizing the necessity of intrinsic pipeline and environmental parameters and individual risk concepts.

The third category focuses on gas pipeline risks for specific purposes, including simulating urban gas pipeline risks, quantitatively assessing high-consequence areas, and evaluating the seismic risks of natural gas networks during earthquakes. Studies in this category, such as Li et al.^18^, Yin et al.^19^ and Nieh and Lin^20^, offer unique insights into the management of gas pipeline risks for specific scenarios and locations. This study focuses on assessing the risk associated with urban gas pipelines in Bojnord, with a particular emphasis on understanding the dynamics of population distribution, demographic characteristics, and building structures. Through the integration of geospatial analysis techniques, hazard modeling, and vulnerability assessment, we aim to gain valuable insights into the spatial and temporal variations in risk exposure to gas pipeline hazards.

Bojnord City was selected as the study area because of incidents involving urban gas pipelines in this region, with recent accidents caused by gas leaks and hazards associated with municipal gas pipelines. In 2016, a gas leak explosion in the Jomhouri neighborhood resulted in two individuals receiving serious burns. Another explosion on Javadiyeh Street in 2017 injured six people, and an explosion in the Golestan and Baharestan neighborhoods in 2018 resulted in both human and financial losses. The city is an important center for industry and commerce, with various businesses and factories utilizing gas pipelines for their operations. Furthermore, Bojnord is situated in a geographically complex region in the foothills of Aladagh, Kopeh Dagh, and Alborz, which adds to the challenges of pipeline management and maintenance. This demonstrates the need for a day–night population model to address the risks of urban gas pipelines and enable the implementation of appropriate safety measures.

## Study area and datasets

### Study area

**Figure 1:**
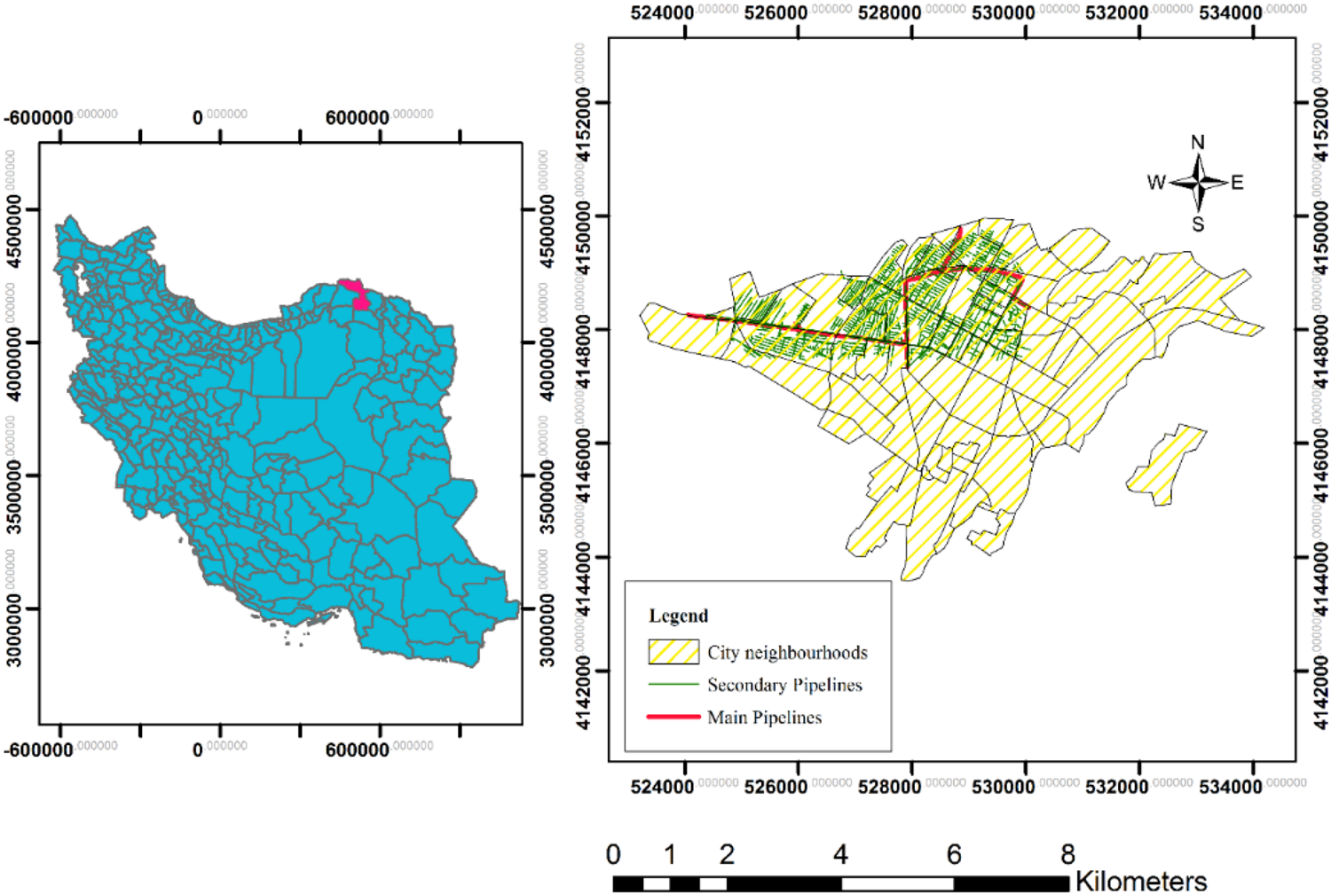
Maps of (**a**) Bojnord City in Iran and (**b**) the study area, city neighborhoods, and the pipeline locations (Map generated using ArcGIS 10.5).

Bojnord City is located in the North Khorasan Province, Northeastern Iran (57° 20′ longitude and 37° 28′ latitude). It has an area of 36 km^2^ and a population of 228,931, including 67,335 households, according to the latest general population and housing census of the Statistics Center of Iran in 2016. Figure 1 shows the location of Bojnord and the gas lines in the study area. Owing to the lack of access to gas pipeline information for the entire city, a hazard map was prepared for Regions 1 and 2. These areas are located in the northern and western areas of Bojnord City and are often used for residential, industrial, and commercial purposes.

### Datasets

Table 1 lists the primary datasets used in this study, which comprises land use, population, urban gas pipeline location information, pipeline parameters, and failure rate. The land use data was extracted from a satellite-based land use layer from 2004 provided by the Municipal Information and Communication Technology Organization. Residential areas included single-family homes, apartments, and other types of housing. Workplaces included offices, factories, and shops. Education facilities included schools, universities, and other institutions. Clinics and hospitals are healthcare facilities providing medical services to the public. Tourist accommodations included hotels, resorts, and other types of tourist lodging. The data on the population size and age structure of each residential district and neighborhood from the latest (2016) population and housing census were obtained from the Statistics Centre of Iran. Table 2 presents the parameters of the gas pipelines investigated in this study. Descriptive information on the gas pipelines in 2015 from the North Khorasan Gas Transmission Company included the pipe type (i.e., material used in the pipeline, such as steel, cast iron, or plastic), gas pressure inside the pipe (i.e., pressure at which the gas is transported through the pipeline), pipe diameter (i.e., internal diameter of the pipeline), and pipe length (i.e., distance covered by the pipeline). This information is important for assessing hazards associated with pipelines.

**Table 1:** Datasets used in the study.

Dataset	Source	Year	Data type	Data description
Land use	Municipal Information and Communication Technology Organization	2004	Vector polygon	Residential areas, workplaces, educational facilities, kindergartens, clinics, restaurants, hospitals, tourist accommodations, road networks, and traffic counting stations, building age, material types, building area, number of floors
Population	Statistics Center of Iran	2016	Table	Population size, age structure of each residential district
Gas pipeline	North Khorasan Gas Transmission Company	2015	Vector polyline	Pipeline location and descriptive information: pipe type, gas pressure inside, pipe diameter, and pipe length
Past gas accident	North Khorasan Gas Transmission Company	2015	Table	Information about past incidents and their causes

**Table 2:** Example of the studied pipeline parameters.

Line ID	Diameter (cm)	Pressure (psig)	Pipe material	Pipe type	Pipe length (m)
1	30.48	250	Steel	Feeder	1344
5	30.48	250	Steel	Feeder	599
10	5.08	60	Steel	Distribution	52
15	5.08	60	Steel	Distribution	48

The failure rate was calculated using empirical formulas based on information gathered by the European Gas Pipeline Incident Data Group (EGIG). Historical data are typically used to estimate the likelihood of a pipeline failure. However, the probability of failure can vary significantly across different sections of a pipeline and is influenced by factors such as the design, construction, maintenance, and environmental conditions. This probability was measured as the number of failures per unit pipe length per year assuming uniform conditions along the section of interest. Table 3 shows the proportions of various causes of pipeline failure per 1000 km, as reported by the EGIG.

**Table 3:** Pipeline failure frequency categorized based on failure cause and leak size^21^.

Cause	Failure frequency per 1000 km/y			
	Rupture	Hole	Pinhole/crack	Unknown
External interference	0.0058	0.0195	0.0166	0.0007
Corrosion	0.0000	0.0007	0.0353	0.0014
Construction defects	0.0022	0.0014	0.0224	0.0007
Hot tap	0.0000	0.0014	0.0043	0.0000
Ground movement	0.0065	0.0079	0.0065	0.0014

## Methods

**Figure 2:**
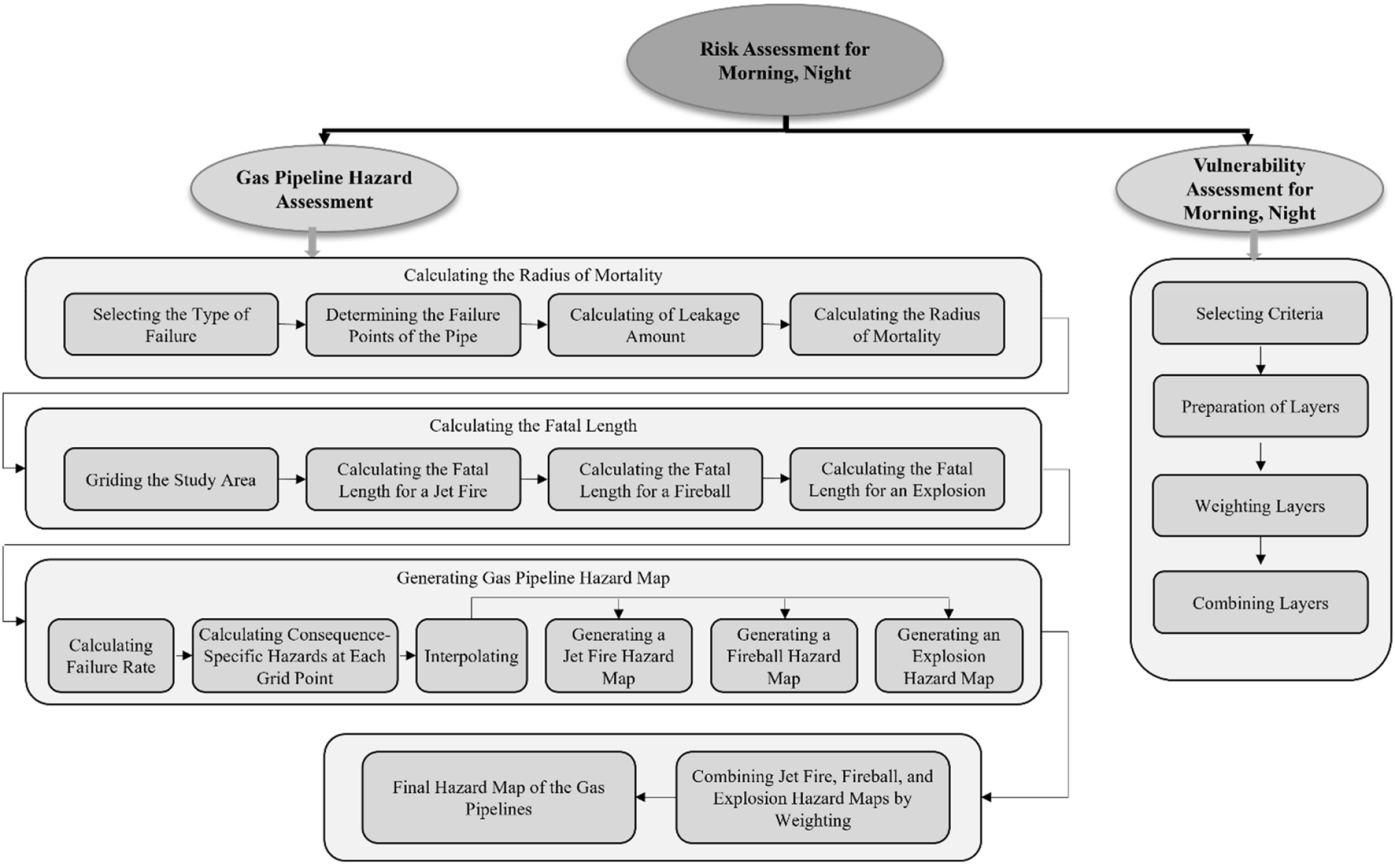
Process for gas pipeline risk assessment for both night and morning period.

This study aims to assess the risk associated with gas pipeline hazards for both night and morning in Bojnord. The methodology employed for this assessment involves measuring both hazard and vulnerability. To assess hazards, the following steps were undertaken. The study area was gridded to prepare a hazard map. The hazards of gas pipelines for grid points were calculated using the intrinsic parameters of gas pipelines and some environmental parameters (“Urban gas pipeline hazard assessment” section). Finally, the degree of hazard at these points was generalized to all areas using interpolation tools. In addition to assessing hazards, vulnerability criteria were incorporated into the analysis. One such criterion is the dynamic nature of the population. The vulnerability assessment accounts for variations in population density and activity levels during both night and morning periods, recognizing that these factors can influence the potential impact of gas pipeline hazards on the community. Figure 2 shows the research steps used to perform the gas pipeline risk assessment, specifically addressing the hazards and vulnerabilities associated with both night and morning periods.

### Day–night vulnerability assessment

In this section, we provide a detailed description of the approach used to evaluate how susceptible the study area is to risks associated with gas pipelines. This assessment is conducted with a focus on understanding vulnerability during both day and night periods, recognizing that factors influencing vulnerability may vary depending on the time of day. The methodology encompasses several critical stages to ensure a comprehensive evaluation. Firstly, we discuss the process of selecting specific criteria that are pertinent to understanding vulnerability in relation to gas pipeline hazards. These criteria are carefully chosen based on their relevance to the study objectives and their potential impact on vulnerability. Secondly, we elaborate on the procedure of weighing these criteria, which involves assigning relative importance or significance to each criterion. This step is essential for ensuring that the assessment accurately reflects the relative influence of different factors on vulnerability. Finally, we describe the process of vulnerability mapping, wherein the selected criteria are combined and analyzed spatially to generate maps that depict the distribution and intensity of vulnerability across the study area. By following these key steps, we aim to develop a comprehensive understanding of vulnerability to gas pipeline risks, taking into account variations between day and night periods.

#### Selection of criteria

Criteria for vulnerability assessment are chosen based on research objectives and available data. The vulnerability assessment in this study involves categorizing parameters into distinct groups, including demographic, structural, and environmental factors, alongside proximity to key infrastructure sites. Structural parameters encompass characteristics related to the built environment, particularly within urban blocks. These factors include age of buildings, building area, material type, number of floors, and proximity to transportation networks. These structural attributes play a significant role in determining the resilience of structures to potential gas pipeline hazards. Within the social category, demographic variables such as age structure, gender composition, and population density of urban blocks are considered. The dynamic nature of the population, reflected in Day–Night maps capturing variations in population density and activity levels, provides crucial insights into vulnerability patterns. Understanding the demographics of the population is essential for comprehensively assessing susceptibility to gas pipeline risks. Additionally, environmental factors such as distance from gas stations and city gas lines are taken into account, as these infrastructures may pose additional risks in the event of an incident. By considering these diverse criteria, the vulnerability assessment aims to provide a comprehensive understanding of the factors contributing to the susceptibility of the study area to gas pipeline risks.

#### Weighting of criteria layers

In vulnerability assessment, it’s essential to understand the relative importance of different criteria in contributing to overall vulnerability. The Analytic Hierarchy Process (AHP) method provides a structured approach to assign weights to various criteria layers based on their significance. The AHP method involves breaking down the complex decision-making process into a hierarchy of criteria and sub-criteria. Decision-makers then compare pairs of criteria and sub-criteria, assigning relative importance or priority to each pair based on their judgment. This pairwise comparison process is facilitated through numerical scales, allowing for the quantification of subjective judgments^22^. Once all pairwise comparisons are completed, the AHP method calculates priority weights for each criterion relative to others. These weights reflect the relative importance of each criterion in influencing vulnerability.

#### Preparation and combination of criteria layers

Criteria layers are developed based on selected parameters identified for vulnerability assessment. This phase involves collecting pertinent data and transforming it into spatial layers that represent each criterion. Through this process, we ensure that relevant aspects of vulnerability are captured spatially across the study area.

Following the preparation of criteria layers, the next step involves combining them to generate comprehensive vulnerability maps. This integration allows us to account for the varying degrees of influence that each criterion has on vulnerability. By utilizing the weights assigned through the AHP method, the criteria layers are combined to produce vulnerability maps for both night and morning populations. Through overlaying and synthesizing these layers, we obtain vulnerability maps for day–night periods.

#### Day–night population distribution

The urban landscape experiences a significant transformation during nighttime hours that is characterized by a marked reduction in activity and a sense of quiet orderliness. This transition occurs because most residents retreat to their homes or apartments, except for night shift employees. Because the population tended to remain steady during the night, the floating population was omitted from the nighttime population data for this study. Many human activities, such as work, study, leisure, and shopping, occur during the day. The daily population is generally classified into four groups: (1) working population; (2) resident population; (3) students; and (4) random population, such as customers, patients, and tourists, based on the classification method of Qi et al.^23^. The population obtained from the census data was assigned to these four categories of daytime population, which pertains to specific building-level data.

Lwin and Murayama^24^ introduced two techniques for the population estimation of a building: a metric area technique that does not require knowledge of the number of floors and a volumetric technique that requires knowledge of the number of floors. The overnight population of residential buildings was estimated via the volumetric method using Eqs. (1) and (2):

1 $$Pop_{B\left( n \right)} = Pop_{C\left( k \right)} .A_{B\left( n \right)} .F_{B\left( n \right)} /\left( {\mathop \sum \limits_{i = 1}^{M} A_{B\left( i \right)} .F_{B\left( i \right)} } \right)$$

2 $$Pop_{nighttime} = \mathop \sum \limits_{k = 1}^{K} Pop_{C\left( k \right)} = \mathop \sum \limits_{n = 1}^{N} Pop_{B\left( n \right)}$$

where *Pop*_*B(n)*_ is the population of residential building *n, Pop*_*c(k)*_ denotes the resident population in the census tract, *A*_*B(i)*_ represents the area of residential building *i*, *F*_*B(i)*_ represents the number of floors in residential building *i*, *M* denotes the number of residential buildings within the neighborhood, *N* represents the total number of residential buildings in the case study, *K* denotes the total number of census tracts in the case study, and *Pop*_*nighttime*_ represents the total night residential population. The daily population was estimated from land use data using Eq. (3).

3 $$Pop_{daytime} = \mathop \sum \limits_{n = 1}^{N} Pop_{Bd\left( n \right)} + \mathop \sum \limits_{m = 1}^{M} Pop_{W\left( m \right)} + \mathop \sum \limits_{l = 1}^{L} Pop_{S\left( l \right)} + \mathop \sum \limits_{k = 1}^{K} Pop_{CT\left( k \right)}$$

where *Pop*_*daytime*_ is the total resident population during the day, *Pop*_*Bd(n)*_ is the residential building during the day, *Pop*_*W(m)*_ is the working population, *Pop*_*S(l)*_ denotes the population in educational areas, and *Pop*_*CT(k)*_ represents the number of customers, patients, and tourists within green and recreational areas, shops, markets, or hospitals. During the day, the resident population consists mostly of elderly people and children. The population of the residential areas during the day was calculated using Eq. (4):

4 $$Pop_{Bd\left( n \right)} = Pop_{Cd} .A_{B\left( n \right)} .F_{B\left( n \right)} /\left( {\mathop \sum \limits_{i = 1}^{M} A_{B\left( i \right)} .F_{B\left( i \right)} } \right)$$

where *Pop*_*Cd*_ is a resident population that includes the elderly over the age of 65 and children under the age of six in each census tract and *Pop*_*Bd(n)*_ is the population of children and elderly during the day. Furthermore, the working population was calculated using the following equation:

5 $$Pop_{W\left( m \right)} = \left\{ {\begin{array}{*{20}c} {\mathop \sum \limits_{i = 1}^{I} E_{i} , if \mathop \sum \limits_{i = 1}^{I} E_{i} < Limit_{Pop} } \\ {Limit_{Pop} , if \mathop \sum \limits_{i = 1}^{I} E_{i} \ge Limit_{Pop} } \\ \end{array} } \right.$$

where *TE*_*i*_ is the number of employees in the company or department. *Limit*_*Pop*,_ which represents the largest number of employees per building, is an empirically assessed value that uses the experience of experts and the number of people assigned to industrial and office properties utilized to constrain the maximum workplace population. Equation (5) can also be used to calculate the student population *Pop*_*S(l)*_. Finally, the population of any green space, market, or hospital was calculated using Eq. (6):

6 $$Pop_{CT\left( k \right)} = \left( {\mathop \sum \limits_{i = 1}^{I} Pop_{Sample\left( i \right)} /\mathop \sum \limits_{i = 1}^{I} A_{Sample\left( i \right)} } \right).A_{CT\left( k \right)}$$

where *Pop*_*Sample(i)*_ is the population sample *i* of hospitals, markets, and green spaces, *A*_*Sample(i)*_ denotes the area of sample *i* estimated using sampling and field observations, and *A*_*CT(k)*_ and *Pop*_*CT(k)*_ represent the area and population of the market, green space, or hospital *k*, respectively^25,26^. This is used to determine the mean population density of markets, green spaces, and hospitals. We selected ten green spaces, 15 markets, and three hospitals for this purpose. Population samples from these areas were collected over seven days, specifically five workdays and two weekends. Moreover, the use of surveys and their subsequent validation against real-world data played a pivotal role in enhancing the accuracy and reliability of the population estimates. Modeling was performed by selecting the target time for the population. The entire neighborhood was processed with respect to this time, and the necessary number of individuals was assigned to destination activities, such as workplaces, schools, traveling, and residences.

### Urban gas pipeline hazard assessment

#### Radius of mortality

The first step in the analysis phase involves the determination of the failure type that may occur in a gas pipeline. These failures can be classified into two primary categories: puncture and full-bore rupture. Punctures are further categorized based on the size of the holes created during failure. These size categories included small holes (diameter ≤ 2 cm), medium-sized holes (diameter > 2 cm but smaller than the pipe diameter), and large holes (diameter > pipeline). The distribution of full-bore ruptures and punctures based on previous studies was found to be in the ratio 0.25:0.75^27^. In this study, we focused exclusively on the impacts of full-bore ruptures, encompassing both the first and second types.

Following the identification of the failure type, the exact locations of the pipeline failures were determined. The next critical step involves calculating the rate at which the gas is emitted from the failure site. This calculation is highly dependent on the failure type and is calculated using Eqs. (7) and (8). These equations were designed to calculate the gas emission rates under different flow conditions and are applicable to sonic and subsonic flows, while only Eq. (8) is utilized for subsonic flow. In these equations, *Q* represents the mass flow rate of gas emission, *A* is the pipe gap area (m^2^), *M* denotes the molecular weight of the gas, *R* is the gas constant (8.314 J/mol K), *T* represents the in-pipe gas temperature (K), *k* is the adiabatic index, *P*_*0*_ is the ambient pressure (Pa), and *P*_*1*_ represents the in-tube pressure.

7 $$Q = C_{0} AP_{1} \sqrt {\frac{KM}{{RT}}\left( {\frac{2}{k + 1}} \right)^{{\frac{k + 1}{{k - 1}}}} } \quad {\text{sonic}}$$

8 $${\text{Q}} = {\text{C}}_{0} {\text{AP}}_{1} \sqrt {\frac{{2{\text{M}}}}{{{\text{RT}}}}\left( {\frac{{\text{k}}}{{{\text{k}} - 1}}} \right)\left[ {\left( {\frac{{{\text{P}}_{0} }}{{{\text{P}}_{1} }}} \right)^{{\frac{2}{{\text{k}}}}} \_\left( {\frac{{{\text{P}}_{0} }}{{{\text{P}}_{1} }}} \right)^{{\frac{{{\text{k}} + 1}}{{\text{k}}}}} } \right]} \quad {\text{subsonic}}$$

Different consequences, such as the spread of toxic gases, sudden fires, jet fires, fireballs, pool fires, and explosions, may occur in the pipe after failure in the lines. Spreading toxic gases can result in health hazards; sudden fires can damage buildings and lead to injuries or fatalities; jet fires can cause structural damage and immediate danger; fireballs are intense and destructive; pool fires can engulf structures; and explosions can cause significant damage, including structural collapse. The extent of damage and population loss in each case varies based on factors such as the type and volume of gas involved, building proximity, construction, and emergency response measures. In particular, the consequences of fireballs, jet fires, and explosions due to line failures are discussed in this study.

The hazard range of pipelines is determined by calculating the radius of mortality at three levels, 1%, 50%, and 99%, using Eqs. (9) to (12) ^14,17,28^.

9 $$r_{jet,99} = 3.891\sqrt Q , r_{jet,50} = 5.498\sqrt Q , r_{jet,1} = 7.767\sqrt Q$$

10 $$\frac{{\left( {r_{Fireball,99} } \right)^{\frac{4}{3}} }}{{m^{1.106} }} = 2.855, \frac{{\left( {r_{Fireball,50} } \right)^{\frac{4}{3}} }}{{m^{1.106} }} = 4.518, \frac{{\left( {r_{Fireball,1} } \right)^{\frac{4}{3}} }}{{m^{1.106} }} = 7.149$$

11 $$\frac{{r_{Explosion, 99} }}{{\sqrt[3]{{m_{TNT} }}}} = 2.855, \frac{{r_{Explosion, 50} }}{{\sqrt[3]{{m_{TNT} }}}} = 2.861, \frac{{r_{Explosion,1} }}{{\sqrt[3]{{m_{TNT} }}}} = 3.017$$

12 $$m_{TNT} = \frac{{m_{d} \Delta H_{d} }}{{Q_{TNT} }}$$

where *r*_*jet*_*,*_*50*_*, r*_*jet*_*,*_*99*_*,* and *r*_*jet*_*,*_*1*_ are the blast radii of death for mortality levels of 99%, 50%, and 1%, respectively, *r*_*Fireball*_ is the lethal radius of the fireball, *m* is the gas mass, *r*_*Explosion*_ is the lethal radius of the explosion, and m_d_ is the gas mass of the explosion. In Eq. (9), we computed the radius of mortality for jet fire scenarios considering different mortality levels. Equation (10) calculates the lethal radius for fireball consequences with similar considerations for various mortality levels. Finally, Eq. (11) calculates the lethal radii in the event of an explosion.

#### Fatal length

The critical phase of our analysis focused on determining the fatality lengths associated with various hazards, including jet fires, fireballs, and explosions. The fatal length is a pivotal parameter that allows us to understand the extent of the risk and potential consequences of a gas pipeline failure. To conduct this crucial assessment, we first established a grid for the study area consisting of numerous points, each representing a specific location within the area under investigation. Grids are fundamental to hazard analysis because they allow the evaluation of the influence of gas pipeline failures at discrete points within the study area. A systematic grid of points covering the entire study area was created. Each grid point is strategically located to ensure comprehensive coverage. This grid formed the basis of our hazard assessment, allowing us to assess the impact of gas pipeline failures at specific geographic locations.

Three specific mortality levels were considered: 1%, 50%, and 99%. For each of these levels, we employed Eq. (13) for precise calculation^14,17,29^, offering insights into the fatal length parameters and enabling us to accurately gauge the potential risks and outcomes associated with these hazards. Parameters *l*_*jet fire,100_99*_, *l*_*jet fire,99_50*_, *l*_*jet fire,50_1*_, *l*_*fireball,100_99*_, *l*_*fireball,99_50*_, *l*_*fireball,50_1*_, *l*_*explosion,100_99*_, *l*_*explosion,99_50*_, and *l*_*xplosion,50_1*_ were instrumental in calculating the fatal length for these hazards. These values, when combined with the grid-based approach, provide a comprehensive understanding of the extent of the hazards at each grid point. This information is invaluable for developing precise hazard assessment metrics. Finally, three values of the death length, which were due to the consequences of fireballs, jet fires, and explosions, were obtained for each point of the grid using Eqs. (14), (15), and (16). The fatal length, denoted as L and h, is the distance between the grid point and the fault location of the pipeline.

13 $$L_{i,100\_99} = 2\sqrt {r_{99}^{2} - h^{2} } ,L_{i,99\_50} = 2\sqrt {r_{99}^{2} - h^{2} } ,L_{i,50\_1} = 2\sqrt {r_{99}^{2} - h^{2} }$$

14 $${\text{L}}_{{{\text{Fatal Length}},{\text{ jet fire}}}} \approx {\text{l}}_{{{\text{jet fire}},{ }100\_99}} + 0.805{\text{l}}_{{{\text{jet fire}},99\_50}} + 0.172{\text{l}}_{{{\text{jet fire}},50\_1}}$$

15 $${\text{L}}_{{{\text{Fatal Length}},{\text{ fireball}}}} \approx {\text{l}}_{{{\text{fireball}},{ }100\_99}} + 0.831{\text{l}}_{{{\text{fireball}},99\_50}} + 0.161{\text{l}}_{{{\text{fireball}},50\_1}}$$

16 $${\text{L}}_{{{\text{Fatal Length}},{\text{ explosion}}}} \approx {\text{l}}_{{{\text{explosion}},{ }100\_99}} + 0.828{\text{l}}_{{{\text{explosion}},99\_50}} + 0.168{\text{l}}_{{{\text{explosion}},50\_1}}$$

#### Gas pipeline hazard map

A gas pipeline hazard map is a comprehensive tool for understanding and mitigating the risks associated with urban gas pipelines. This map combines multiple steps to provide a holistic view of the hazards and potential consequences, thereby enhancing urban safety planning.

Failure rate calculation: We first determined the failure rate of gas pipelines, which is a pivotal parameter in assessing pipeline safety. These rates, expressed as the frequency of pipeline failures per unit length per year, are used to evaluate the risks associated with each pipeline segment. To calculate these rates, we drew on established empirical formulas using data from the EGIG database (Table 1)^21^, a valuable resource for pipeline safety analyses widely recognized for its comprehensive information^17,30,31^. Notably, these failure rates vary along the pipeline and are influenced by factors such as soil type, design, pipeline age, and depth of cover.

Sequence-specific hazard calculation at each grid point: After establishing the failure rates, we calculated the hazard levels at each grid point within the study area. To achieve this, we incorporated the fatal length at mortality levels 1%, 50%, and 99%. The outcome was a grid-based assessment of the hazard levels, offering a granular understanding of the risk within the study area. The hazard level was calculated by multiplying the failure rate by the data length at each grade point, after calculating the mortality rate for the grid points of the study area.

Interpolation: With the hazard levels determined at the discrete grid points, the next step was to extrapolate this information across the entire study area. This was achieved through a robust interpolation process in which we opted to use the kriging method. Kriging is a geostatistical technique that generates estimated surfaces from scattered data points and their associated z-values. This provides a powerful approach for spatial estimation and prediction, ensuring that the hazard map accurately represents the entire study area.

Individual hazard map generation: Three distinct hazard maps were generated, each corresponding to a specific consequence of gas pipeline failure: jet fire, fireball, and explosion. These individual maps provide detailed insights into the localized hazards associated with each consequence.

Combined jet fire, fireball, and explosion hazard maps by weighting*:* The hazard map of the gas pipelines was prepared by combining the weights of jet fire, fireball, and explosion; the weights of the layers had the same probability of occurrence, which were 0.5, 0.3, and 0.2. According to API data, the probabilities of various accidents occurring after a pipeline rupture are as follows: toxic gas diffusion = 0.8, jet fire combustion = 0.1, fireball combustion = 0.06, and explosion = 0.04. The likelihood of toxic gas diffusion was not considered; therefore, the probabilities for the remaining consequences were adjusted to 0.5, 0.3, and 0.2^32^.

Final gas pipeline hazard map: The culmination of our efforts was the creation of a final hazard map of the gas pipelines. The accuracy and relevance of the map were validated through a meticulous matching process that aligned designated hazard areas with real-world incidents and past events in the study area. This ensured that the map faithfully represented the potential risks faced by urban communities. The entire research process was conducted using ArcGIS, a robust platform that enables accurate spatial analysis and hazard assessment.

### Day–night urban risk assessment

Risk, within the context of this study, is defined as the interaction between two key parameters: hazard and vulnerability. Hazard refers to events occurring at various locations with differing severities and frequencies over time. Vulnerability pertains to the characteristics of urban areas that render their populations susceptible to the impacts of such incidents. Equation (17) illustrates the relationship between these parameters^17^.

17 $$Risk_{Day - Night} = Vulnerability_{Day - Night} *Hazard$$

In the context of day–night risk assessment, it’s imperative to consider the dynamic nature of both hazard and vulnerability factors. The day–night cycle introduces fluctuations in population density, and activity levels, which in turn influence the potential impact of hazards on urban areas. By integrating hazard assessments, which account for the likelihood and severity of incidents occurring during different times of the day, with vulnerability assessments that consider the varying susceptibility of populations to these incidents, a comprehensive understanding of day–night risk can be achieved.

## Results

Our goal was to assess the day–night gas pipeline risk. We first present the results of the vulnerability assessment, followed by the findings of the hazard assessment. Finally, we show the results of the risk assessment for both day and nighttime.

### Day–night vulnerability assessment

**Figure 3:**
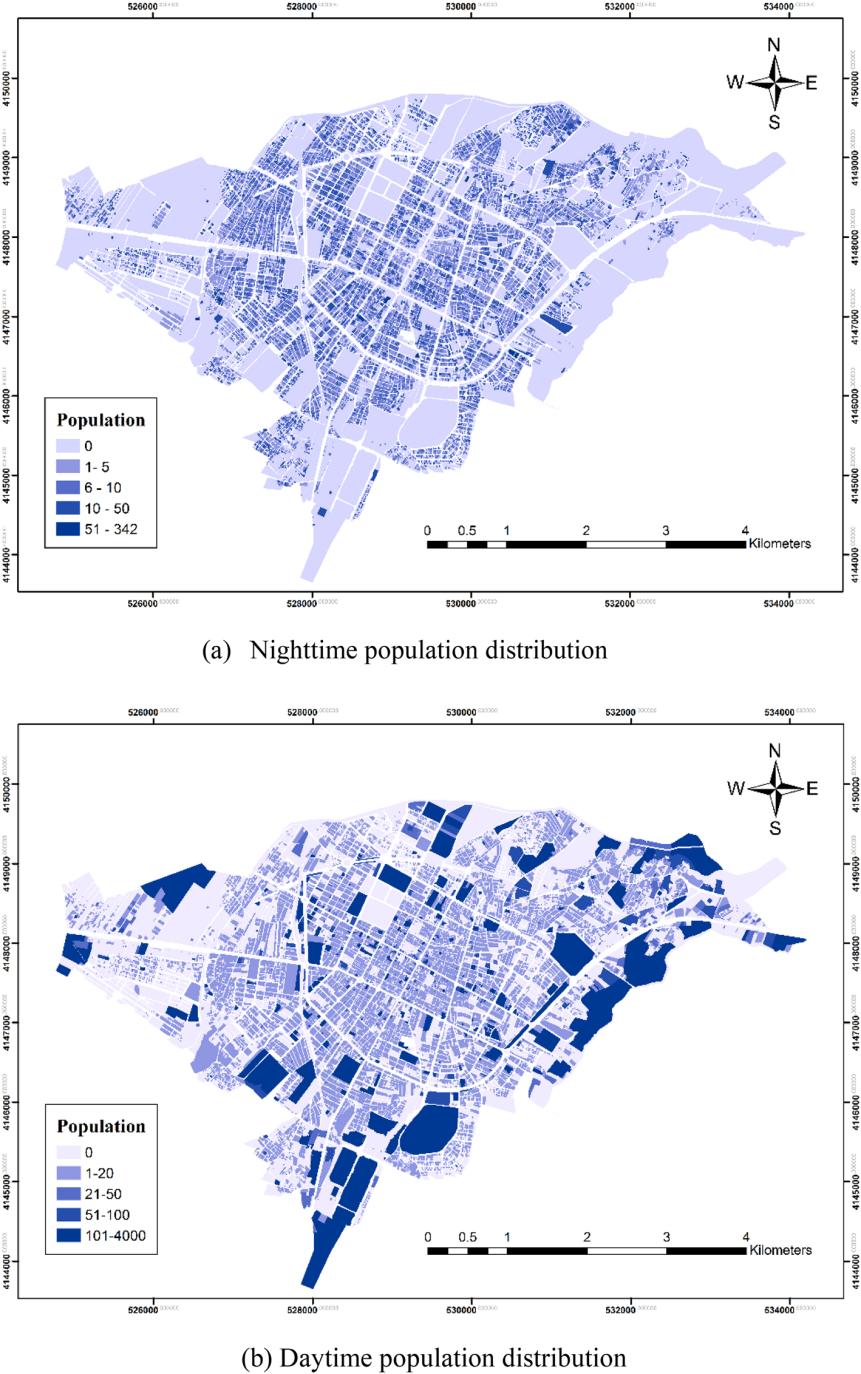
Day and night population distribution maps of Bojnord City (Map generated using ArcGIS 10.5).

For vulnerability assessment, we present the results of the criteria maps, starting with the population distribution for both day and night periods. Figure 3 shows the day and night population distribution maps for the case study. The nighttime population was concentrated in the central residential areas, as represented by the blue regions in Fig. 3a. In contrast, the daytime population significantly varied, expanding from central residential areas to the suburbs, industrial centers, and commercial zones. Figure 3 highlights the multiple daytime population centers, which were predominantly in the educational and commercial areas located in the suburbs and east and south of the city, indicating that these zones influence the daytime population distribution. The day–night population distribution was classified using the Natural Breaks (Jenks) classification method, which identifies natural groupings in the data to highlight meaningful patterns. This classification method was chosen to better represent the underlying patterns in the data and is supported by established GIS practices^33^.

**Figure 4:**
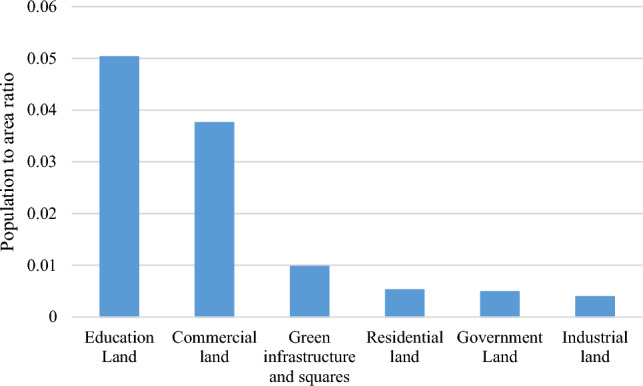
Population-to-area ratio of land use.

Figure 4 shows the population-to-area ratios for different applications. Approximately 45.07% and 33.64% of the city’s population, is educationally and commercially used during the day. The smallest population (3.47%) was associated with industrial use. The residential neighborhood population decreased during the day as people leave their homes to work and study. The population of educational, medical, and working areas increase during the day owing to the arrival of students, patients, and staff, and then declines during the night. The population of tourist places such as parks and green spaces also increases during the day. Almost all the residents stay inside their residential buildings at night. Differences between daytime and nighttime population distributions are attributed to varying daytime and nighttime activities. In addition, the daily laws governing the activity types of urban residents and the distribution of different uses in different urban places cause spatiotemporal variations in the population. Individuals’ activities during the day include rest, work, leisure, excursions, and studying. Figure 3 shows that land use, time, and place influence population distribution.

**Figure 5:**
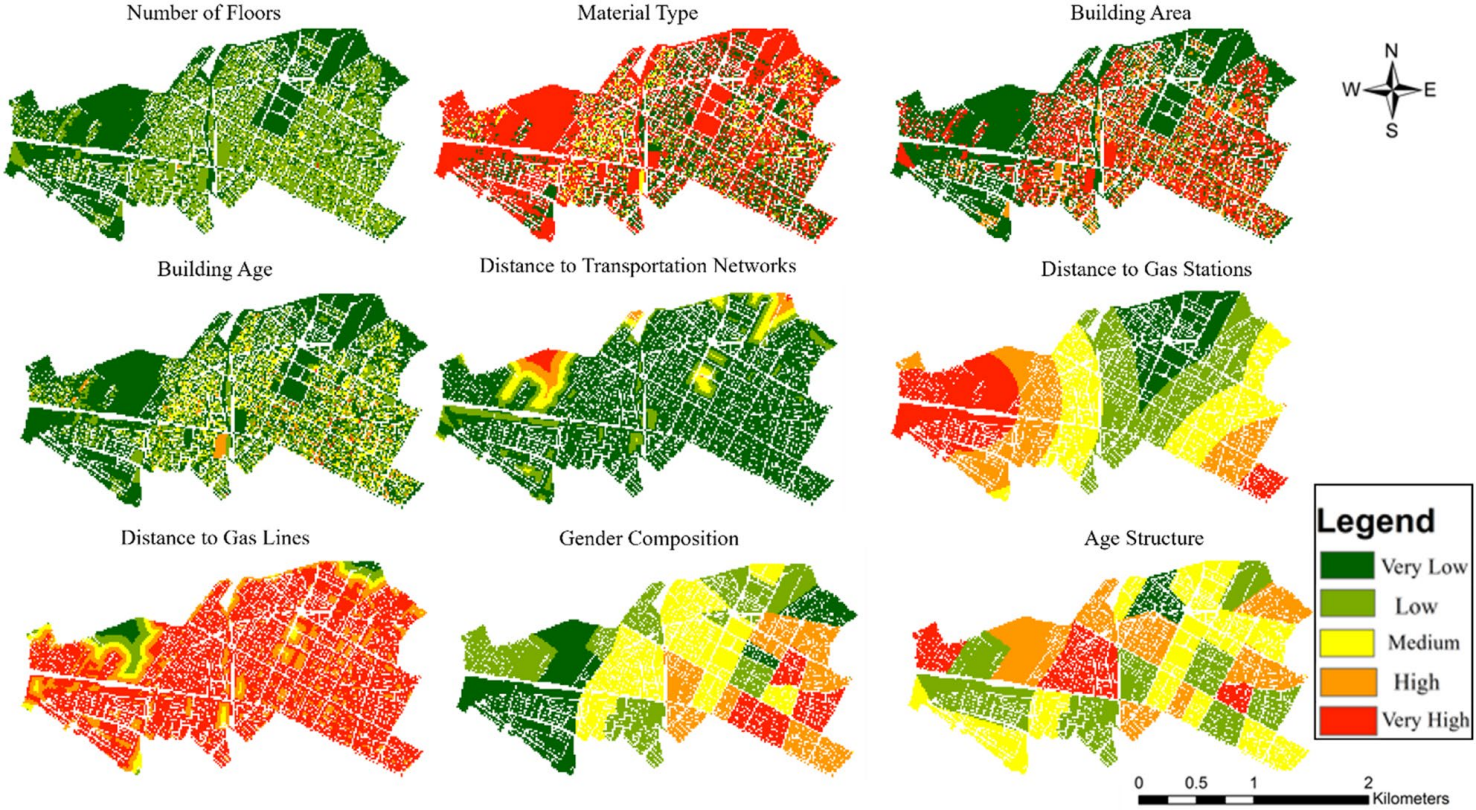
Normalized criteria map (Map generated using ArcGIS 10.5).

In addition to analyzing population distribution, we conducted an in-depth examination of various criteria maps to comprehensively assess vulnerability to gas pipeline hazards in the study area. Figure 5 illustrates the normalized criteria of the study area, showcasing the diverse vulnerability levels across different criteria. The normalized criteria map highlights distinct vulnerability profiles for each criterion within the study area. Notably, areas within the study region exhibit varying levels of vulnerability across different criteria. Upon analysis of the normalized criteria, it becomes apparent that the study area fares relatively well in terms of the number of floors and distance from transportation areas. These aspects show minimal vulnerability. Conversely, the assessment reveals areas of concern for other criteria. Specifically, the study area shows heightened vulnerability in proximity to gas pipelines, age of buildings, and other indicators.

**Figure 6:**
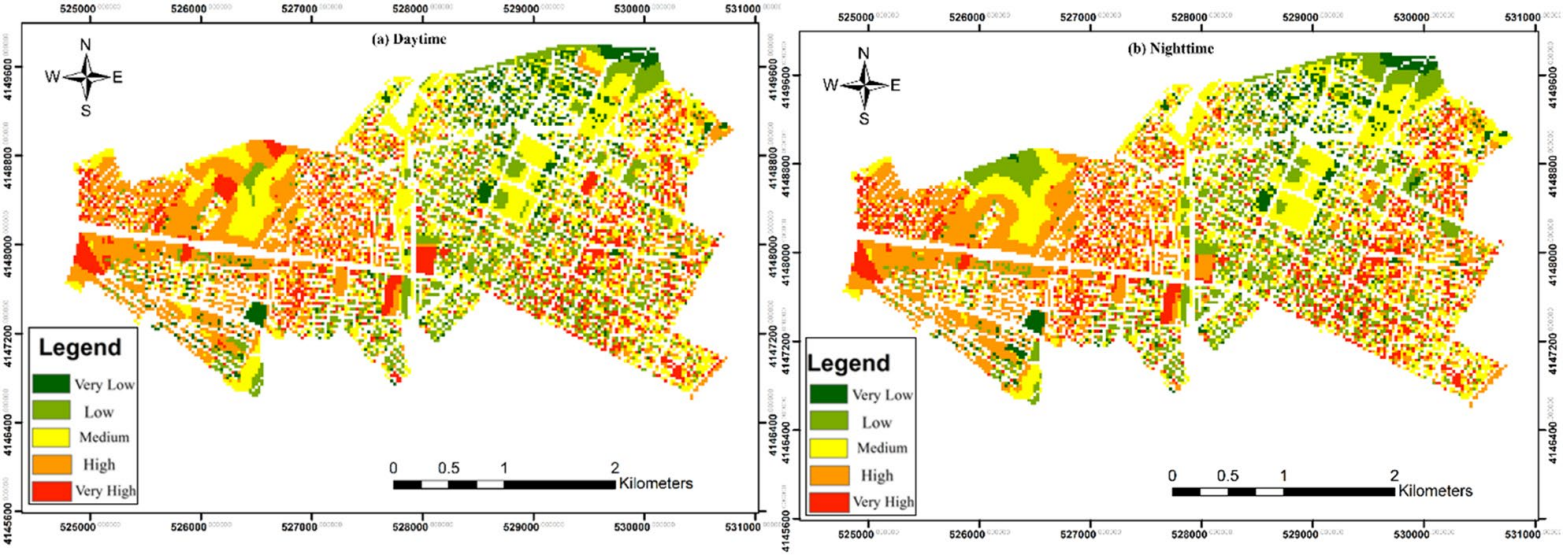
Vulnerability maps (**a**) daytime and (**b**) nighttime periods (Map generated using ArcGIS 10.5).

By mapping vulnerability for each individual criterion and overlaying them with a sum operation, vulnerability maps for both day and night periods were generated (Fig. 6). These maps provide valuable insights into the spatial and temporal variations in vulnerability to gas pipeline hazards. Figure 6a illustrates the vulnerability during the daytime. Analysis of this map reveals that most parts of Regions 1 and 2 exhibit heightened vulnerability, indicating areas of significant concern. However, some areas in the central and northeast regions demonstrate relatively lower vulnerability, suggesting better resilience to gas pipeline hazards. Similarly, Fig. 6b displays the vulnerability during the nighttime period. While this map exhibits a similar pattern to Fig. 6a, notable differences are observed. These differences underscore the influence of population distribution on vulnerability during different periods. The variations in vulnerability between day and night periods highlight the dynamic nature of urban risk and the importance of considering temporal factors in risk assessment and management strategies.

### Hazard assessment

**Figure 7:**
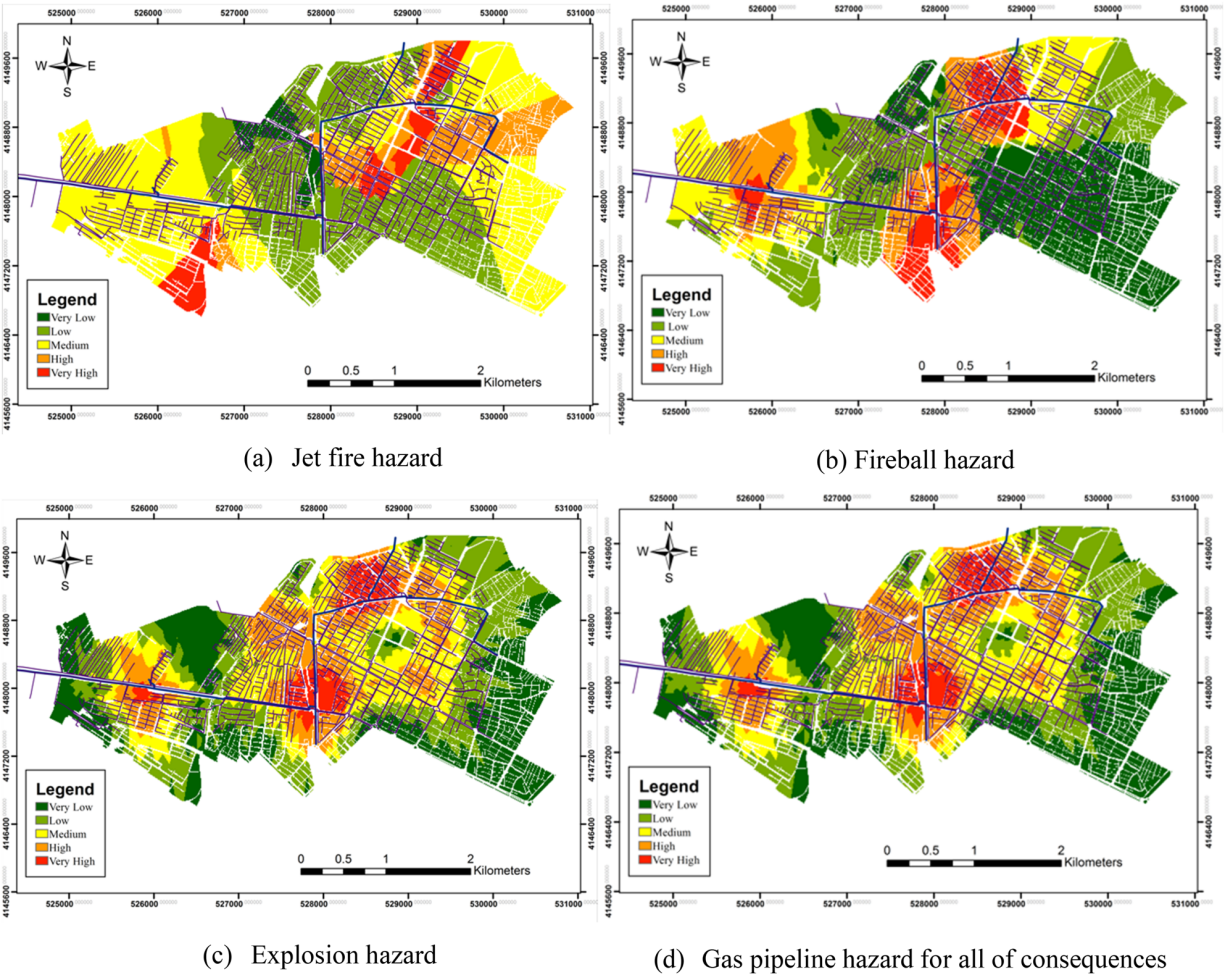
Bojnord gas pipeline hazard map for jet fires, fireballs, and explosions (Map generated using ArcGIS 10.5).

**Figure 8:**
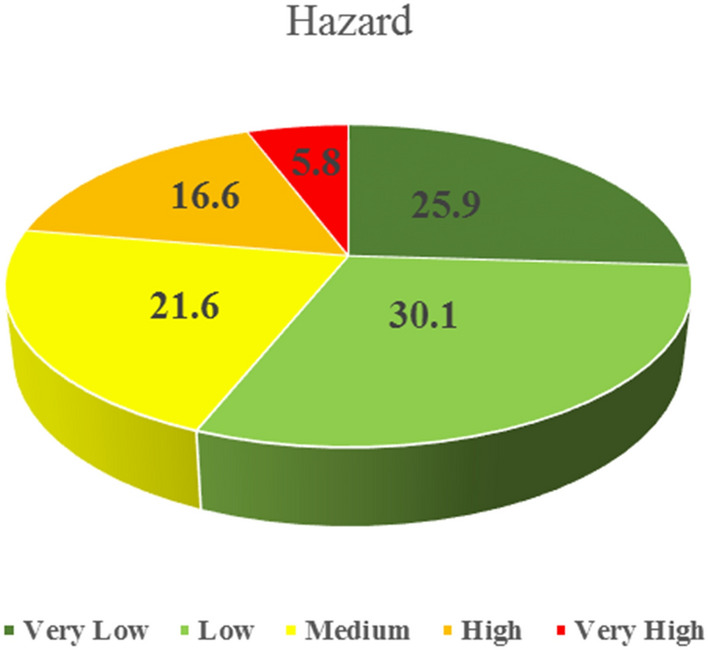
City area percentages under the five hazard classes.

Physical effects, such as the release of toxic gases; fires, including jet fires, fireballs, pool fires, and sudden fires; and explosion pressure, are some of the consequences of pipeline hazards. Only the consequences of jet fires, fireballs, and explosions were considered in this study due to the nontoxicity of methane gas. Figure 7 shows the gas pipeline hazard maps for jet fires, fireballs, and explosions, and the final hazard map. The hazard for fireballs is higher than that of jet fires and explosions owing to its high lethal radius. The danger level at the intersection of the main lines was higher than that in other areas owing to high gas pressure. Furthermore, the hazard level is much higher in the northern, southern, and western regions due to the intersecting main gas pipelines and high sub-pipeline density compared with other locations. The areas allocated for the five very low to very high hazard categories are shown in Fig. 8, with 5.8% and 16.6% of the region having very high and high hazard levels, respectively, and 22.4% of the area was in the dangerous range. The 56% of the area had hazard levels in the low and very low categories and 21.6% of the area was at a medium level.

### Day–night risk assessment

**Figure 9:**
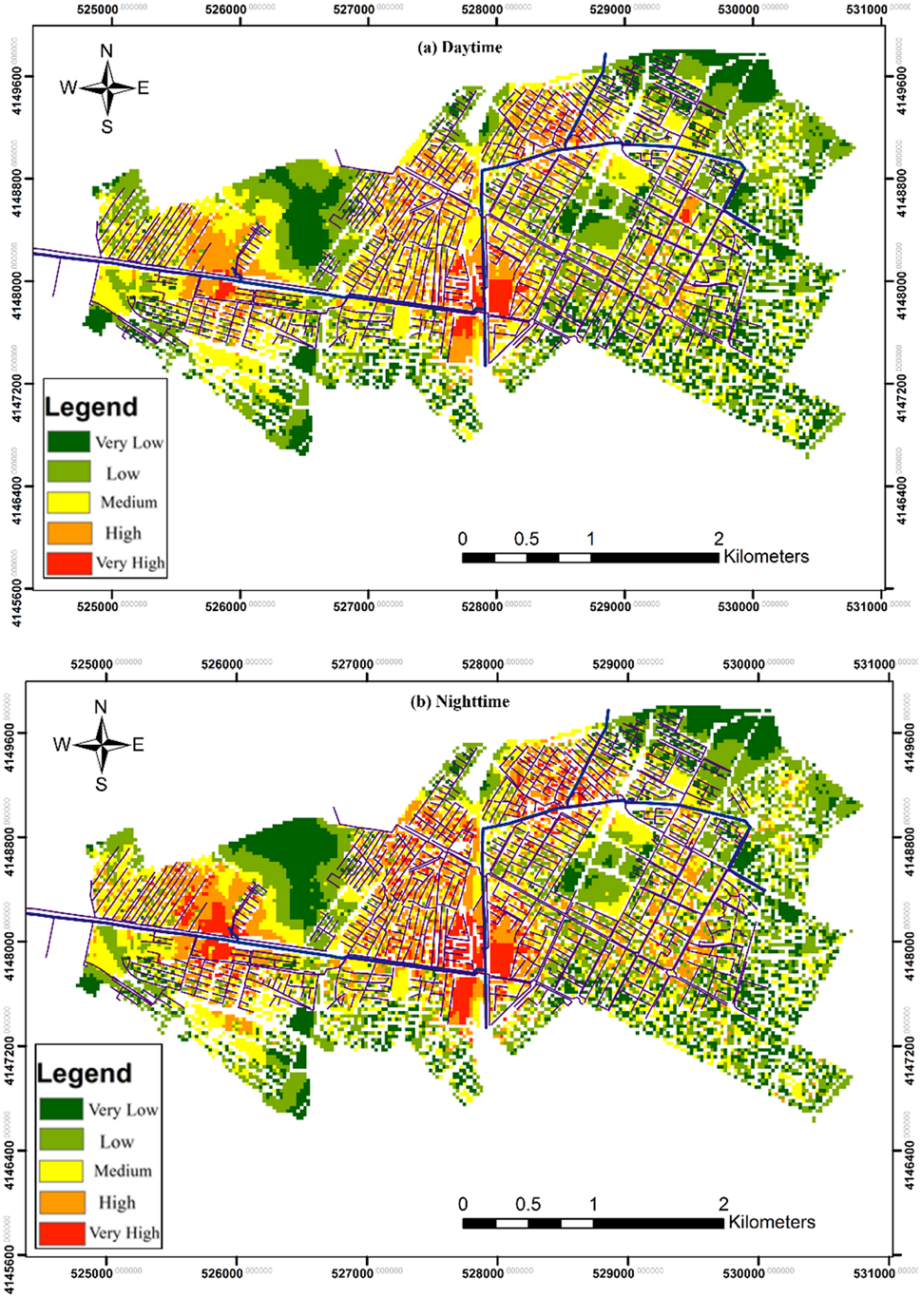
Risk maps (**a**) daytime and (**b**) nighttime periods (Map generated using ArcGIS 10.5).

Figure 9 presents the risk maps for both day and night periods, showcasing the spatial distribution of risk to urban gas pipelines. While Fig. 9a, b generally exhibit similar patterns, differences are observed, underscoring the influence of temporal factors on risk dynamics. These risk maps are derived from the product of hazard and vulnerability maps, integrating various parameters influencing vulnerability within the study area. The resulting risk maps offer valuable insights into the spatial distribution of risk, considering the interplay between hazard exposure and vulnerability factors. Analysis of Fig. 9 reveals that approximately half of the study area exhibits high risk levels during both day and night periods, demonstrating similarities with the patterns observed in vulnerability and hazard maps. This highlights the significance of demographic characteristics and building structures in determining the overall risk profile of an area.

## Discussion

The results of this study demonstrate the importance of considering the dynamics of population distribution, demographic characteristics, and building structures when assessing the risk associated with urban gas pipelines.

Notably, our research revealed distinct patterns in population distribution throughout the day. We found that the daytime population distribution in Bojnord is mainly influenced by educational and commercial centers, whereas the nighttime population is more concentrated in residential areas. The differences in population distribution between daytime and nighttime are due to the various activities that individuals engage in during the day, such as work, study, and leisure. This temporal aspect highlights the unique contribution of our study by acknowledging the dynamic nature of urban populations and their daily routines. The distribution of the urban population is inherently heterogeneous and varies by location and time of day. Understanding these dynamics is paramount for effectively assessing and mitigating risks associated with gas pipelines.

By examining population distribution and conducting an in-depth assessment of vulnerability using multiple criteria maps, we have gained valuable insights into the spatial and temporal variations in vulnerability. Analysis of Fig. 6a indicates that regions 1 and 2 exhibit heightened vulnerability during the daytime, suggesting areas of significant concern. Conversely, some areas in the central and northeast regions demonstrate relatively lower vulnerability, indicating better resilience to gas pipeline hazards. Similarly, Fig. 6b depicts vulnerability during the nighttime period, with notable differences observed compared to the daytime vulnerability map. These differences underscore the influence of population distribution on vulnerability dynamics and highlight the importance of considering temporal factors in risk assessment and management strategies.

The study also analyzed the hazards posed by gas pipelines, specifically the consequences of jet fires, fireballs, and explosions. The results show that such hazards are higher in areas with high gas pressures and intersect the main gas pipelines, which is consistent with the conclusions of previous studies^17,28^. The hazards associated with different consequences shown in Fig. 5d consider the probability of accidents. Evidently, the hazards around certain main pipelines are higher than those around the secondary pipelines. This is because the main pipelines operate at higher pressures than the secondary pipelines. Thus, a correlation exists between the pressure and distance of pipelines and the level of the associated hazards^34^. The danger levels in the very high and high categories were significant, with 22.4% of the area falling into the dangerous range.

**Figure 10:**
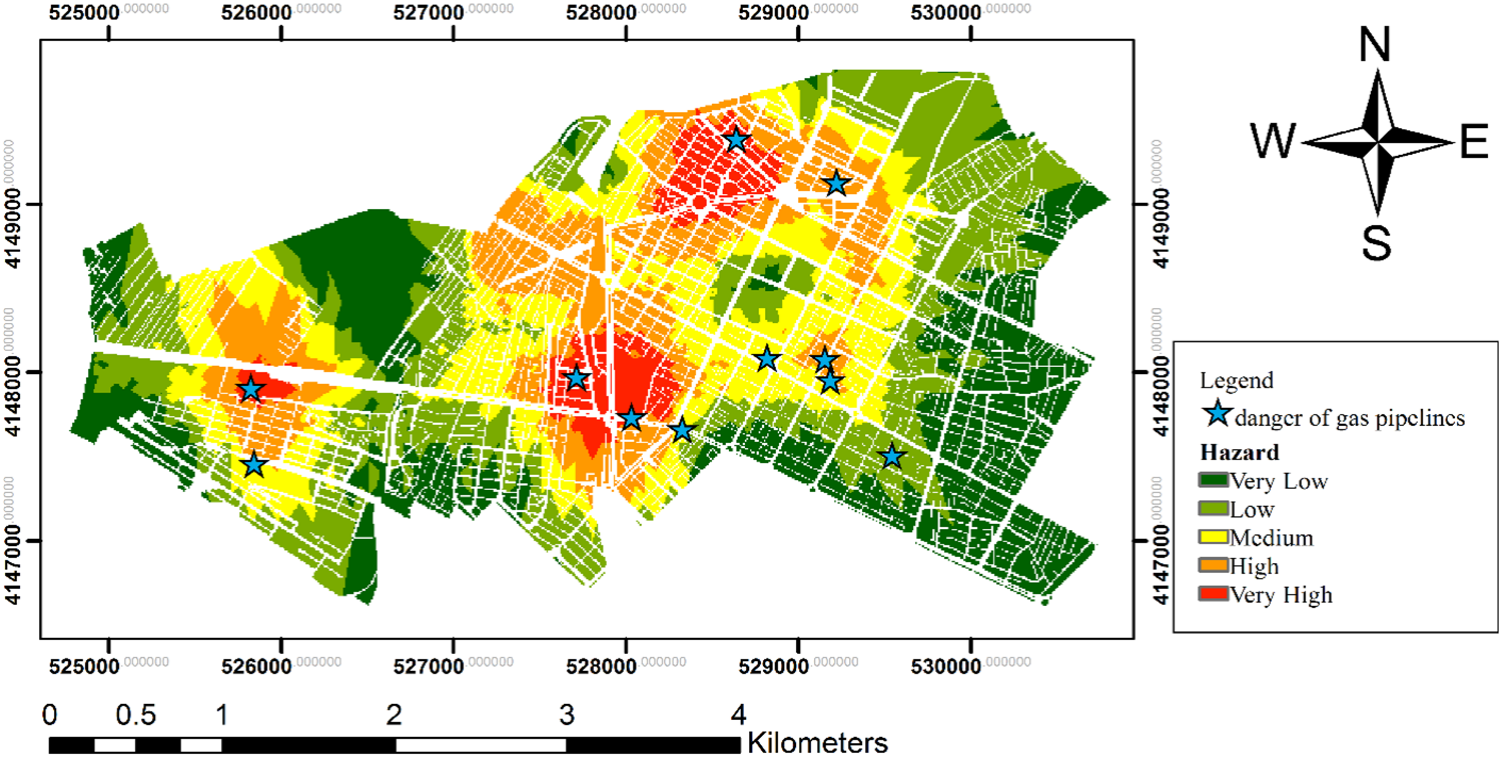
Locations of past pipeline accidents on the hazard map (Map generated using ArcGIS 10.5).

The statistics and specifications of the areas in Bojnord City that have suffered from accidents due to pipeline failures in recent years were obtained from the North Khorasan Gas Company and used to prepare the hazard map to assess and ensure the accuracy of the model. The location of the damaged region is marked on the hazard map. The ratio of past accident areas in each hazard area to the total number of past accidents was obtained, and the locations of past incidents are shown in Fig. 10. The locations of past pipeline accidents were marked as very high, high, or medium danger zones. Of the past accident points, 36.4% were in high hazard areas, 45.4% in high hazard areas, 9.1% in medium hazard areas, and 9.1% in low hazard areas. Based on the total number of past accidents that occurred in areas identified as having medium to high hazards, the accuracy of the estimated model was 90.91%, thereby validating the suitability of the proposed model according to the acceptable accuracy criterion. The findings indicate that the utilized prepared an effective hazard map.

Our research reveals the impact of temporal dynamics on gas pipeline risk estimations. The risk assessment conducted in this study provides critical insights into the spatial distribution of risk to urban gas pipelines during both day and night periods. The risk maps presented in Fig. 9 offer a comprehensive visualization of the potential hazards posed by gas pipelines, considering the interplay between hazard exposure and vulnerability factors. Notably, Fig. 9a, b reveal similar patterns in risk distribution, indicating consistent risk dynamics across different times of the day. However, subtle differences between the day and night risk maps underscore the influence of temporal factors on risk dynamics. These differences may be attributed to variations in population density, land use patterns, and activity levels between day and night periods, highlighting the need for a nuanced understanding of temporal variations in risk assessment.

The risk maps are derived from the product of hazard and vulnerability maps, integrating various parameters influencing vulnerability within the study area. This approach enables a holistic assessment of risk by considering multiple contributing factors, including gas pipeline hazards, demographic characteristics, and building structures. The findings of this study underscore the importance of considering temporal variations in risk assessment, as well as the need to integrate demographic and structural factors into hazard and vulnerability analyses. This approach offers an intuitive yet scientifically sound representation of the distribution of risks that can be easily interpreted by management staff. This modeling technique can help delineate high risk areas and provide valuable insights for decision-making regarding pipeline management and safety by analyzing the population distribution, demographic characteristics, building structures and, characteristics of pipelines and identifying the factors that contribute to variations in risk exposure during different periods.

The limitations of our study should also be discussed. What factors were not considered, and what assumptions were made that might have affected the results? One significant limitation of our study is the omission of dynamic population distribution throughout the day. While we captured population distribution for both day and night periods, we did not account for the fluctuations in population density that occur within the daytime. This limitation may have led to an incomplete understanding of the temporal variations in risk exposure to gas pipeline hazards. Future research endeavors should aim to incorporate real-time population data to capture the full extent of temporal dynamics and their influence on hazard assessment.

## Conclusion

This study offers a comprehensive assessment of the risks associated with urban gas pipelines by considering the dynamics of population distribution, demographic characteristics, and building structures. The findings of this study revealed intriguing patterns. The daytime population distribution was characterized by dispersion, extending from the city center to the suburbs. This dispersion was influenced by the urban structure and growth of commercial centers, offices, agricultural areas, and industrial zones, reflecting the daily travel patterns between residential and work areas. At night, the population tended to concentrate in central areas because of their higher residential density, reinforcing the role of these areas as residential hubs. Furthermore, our analysis of vulnerability using multiple criteria maps reveals spatial and temporal variations in vulnerability to gas pipeline hazards. While certain regions exhibit heightened vulnerability during the daytime, others demonstrate relatively lower vulnerability, indicating varying levels of resilience to gas pipeline hazards.

Our analysis of the impact of fireball hazards highlights the significant dangers posed by gas leakage incidents, which are primarily attributable to their extensive lethal radius. Hazard levels are higher at the intersection of main pipelines owing to the presence of connections and elevated gas pressure. Our research achieved an accuracy rate of 90.91%, thus validating the effectiveness of our hazard assessment methodology.

The risk maps generated in this study, derived from the integration of hazard and vulnerability maps, provide critical insights into the spatial distribution of risk to urban gas pipelines during both day and night periods. While the risk patterns remain largely consistent across different times of the day, subtle differences underscore the influence of temporal factors on risk dynamics, emphasizing the need for a nuanced understanding of temporal variations in risk assessment. What sets our study apart is its unique focus on the dynamics of daytime and nighttime population distributions in relation to gas pipeline risks, which has not been extensively explored in previous research. The study’s findings resonate with Azari and Karimi’s^17^ emphasis on modeling of risk values, aligning with their approach of considering inherent parameters of gas pipelines and environmental conditions. Additionally, in line with Amir-Heidari et al.^15^ and Bariha et al.^16^, who highlight the hazards of gas pipeline failures, our study contributes novel insights by integrating temporal dynamics into risks assessment.

The method proposed in this study can be used in other cities to identify urban areas and populations most affected by urban gas pipeline hazards during specific times. Hazard assessment and modeling of the population is effective for shorter time periods and can be utilized for hourly disaster preparedness and management. Other non-statistical methods can also be used to model spatiotemporal populations.

## Data Availability

The datasets analyzed during the current study are not publicly available, as our adherence to contractual obligations with the source companies restricts the dissemination of the data. Nevertheless, interested parties may obtain access by submitting a formal request to the corresponding author via email at fatemehr937@gmail.com. Provision of the data will be contingent upon securing requisite permissions from the pertinent corporate entities.
